# Body dissatisfaction and low self-esteem in elementary school-aged children: the role of media pressure and trust in parent–child relationships

**DOI:** 10.3389/fpsyg.2023.1228860

**Published:** 2024-01-03

**Authors:** Jolien De Coen, Sandra Verbeken, Lien Goossens

**Affiliations:** Department of Developmental, Personality and Social Psychology, Ghent University, Ghent, Belgium

**Keywords:** body dissatisfaction, self-esteem, media, parent–child relationship, middle childhood

## Abstract

Studies have indicated that the media plays a role in the development of body dissatisfaction in children. Nevertheless, there is limited understanding of the protective factors that may reduce this association, such as the parent–child relationship. Therefore, this study investigates children’s body dissatisfaction and self-esteem and the role of media pressure and a trust in parent–child relationships herein. A sample of *n* = 246 participants (59.8% girls, aged 8–10) was recruited. Children completed self-report questionnaires that assessed body dissatisfaction, self-esteem, media pressure, and trust in parent–child relationships. Results revealed that higher scores on media pressure were linked to increased body dissatisfaction and decreased self-esteem. On the other hand, higher scores on trust in mother and father were associated with lower levels of body dissatisfaction and higher levels of self-esteem. However, the presence of trust in parent–child relationships did not attenuate the impact of media pressure on body dissatisfaction or self-esteem. Further investigation is necessary to gain a deeper understanding of how sociocultural and interpersonal factors interact and contribute to the development of body image problems. While current prevention and intervention programs predominantly focus on the individual, it may be beneficial to place greater emphasis on the family environment.

## Highlights

- Media pressure is associated with more body dissatisfaction and less self-esteem.
- More trust in parent–child relationships is associated with less body dissatisfaction and more self-esteem.
- Both the relationship with the mother, as well as with the father play a role in children’s body evaluation and self-esteem.
- Trust in parent–child relationships did not attenuate the association between media pressure and the outcomes.

## 1 Introduction

A growing body of research indicates that body dissatisfaction is already prevalent during middle childhood (Neves et al., 2017; Paxton and Damiano, 2017; Latiff et al., 2018; World Health Organization, 2020; Navarro-Patón et al., 2021). Sociocultural influences, are primarily targeted as potential risk factors in research on the development of body dissatisfaction (Stice and Whitenton, 2002; McCabe and Ricciardelli, 2005; Cash and Smolak, 2011). This sociocultural perspective on the development of body dissatisfaction has been framed in the Tripartite Influence Model (Thompson et al., 1999). This model posits that three key influence variables - media, peers and parents- influence body dissatisfaction via two mediating mechanisms: internalization of appearance ideals and social comparison. Media is highlighted as one of the main contributors to the mass distribution of beauty ideals (Tiggemann and Slater, 2013; Fardouly and Vartanian, 2016; Neves et al., 2017; Saiphoo and Vahedi, 2019; Faelens et al., 2021). Children’s body image seems to be vulnerable to these harmful media influences since the construction of their self-image is still ongoing, as well as the development of their appearance schema, which includes the beliefs about appearance and the importance one attaches to it (Steinberg, 2018). Experimental (Dittmar et al., 2006; Slater et al., 2017), cross-sectional studies (Anschutz et al., 2009; Tiggemann and Slater, 2014, 2015; Fardouly et al., 2020) and longitudinal (Steinsbekk et al., 2021) have indicated that being exposed to media is associated to an increased level of body dissatisfaction in children Apart from media exposure, subjective aspects of media influence, such as the perceived pressure to conform to beauty ideals imposed by the media, appear to be associated with body dissatisfaction (Roberts et al., 2022). Studies in children and adolescents support this assumption as media pressure predicted an increase in body dissatisfaction and eating problems among both boys and girls (Knauss et al., 2007; De Coen et al., 2021).

However, our understanding of protective sociocultural factors that can potentially mitigate the negative impacts of media influences on children, such as the parent–child relationship, remains limited (Kazdin, 2003). In this study, we will specifically examine the level of trust, which evaluates the extent of mutual respect and trust within the parent–child relationship (Moreira et al., 2017). Attachment theory poses that the early pattern of parental behavior shapes an internal working model containing thoughts, beliefs, expectations, emotions, and behaviors about the self and others. Thus, if caregivers are available and sensitive, children construct positive models of the self and higher levels of self-esteem (Arbona and Power, 2003; Laible et al., 2004). Additionally, adults with insecure attachments, who do not have their interpersonal needs fulfilled, often experience negative feelings about themselves overall, including their bodies (Cash and Smolak, 2011). This connection between insecure attachment and eating disorders in children and adolescents is supported by a review of both cross-sectional and longitudinal studies (Jewell et al., 2016). An insecure attachment was predictive for body dissatisfaction in adolescents (Szalai et al., 2017). In contrast, research has shown that children (Smith et al., 2016) and adolescents (Bearman et al., 2006; De Vries et al., 2019) who reported more positive relationships with their parents demonstrated lower levels of body dissatisfaction. However, there is limited knowledge regarding the specific contributions of the father-child and mother–child relationships. Nevertheless, evidence suggests that both the mother–child and father-child relationships play a (distinct) role in children’s well-being (Bosmans and Kerns, 2015; Keizer et al., 2019). For instance, a longitudinal study involving preadolescent boys and girls discovered that an insecure attachment to the mother significantly predicted an increase in dietary restraint, eating concerns, weight concerns, and shape concerns, while an insecure attachment to the father was linked to the persistence of subjective binge eating episodes in children (Goossens et al., 2012). Although most studies in the field of body image and eating problems predominantly focus on the attachment to the mother (Smith et al., 2016; Siegel et al., 2021; Goslin and Koons-Beauchamp, 2023), this particular study highlights the significance of considering the attachment to the father as well.

Given the encouraging idea that a secure parent–child relationship may promote a healthy body image in children, this raises the question whether a secure parent–child relationship can also protect children’s body image and self-esteem from harmful media influences. From a developmental psychopathological perspective, protective factors are not merely the reverse of risk factors but they alter the strength of the effect of the risk factor (Kazdin, 2003). A secure parent–child relationship may help to attenuate negative emotions caused by media (Cheng and Mallinckrodt, 2009; Hardit and Hannum, 2012; Meeus et al., 2018). Individuals who have secure relationships with their parents tend to develop a more stable and positive self-image. As a result, their self-worth is less reliant on seeking acceptance from others by conforming to societal body ideals (Stein and Corte, 2003). These findings were supported by Cheng and Mallinckrodt (2009) and Hardit and Hannum (2012), who found that female students who had positive relationships with their parents were less likely to internalize appearance ideals, making them less susceptible to sociocultural influences on body image. In a recent cross-sectional study conducted by De Vries et al. (2019), the researchers examined whether a positive mother-adolescent or father-adolescent relationship could moderate the association between media exposure and body dissatisfaction among male and female adolescents aged 12–19 years. These researchers found that media use was positively associated to body dissatisfaction but that this association was weaker for adolescents who reported a more positive relationship with their mother. However, this moderation effect was not found when considering a positive relationship with the father as moderator. These results raise the question whether this buffering mechanism also occurs among elementary school-aged children and whether differences occur depending on the relationship with the mother or the father and depending on the gender of the child. Taken together, the current study aims to shed light on (1) the association between media pressure and body dissatisfaction and self-esteem and (2) the association between trust in parent–child relationships and body dissatisfaction and self-esteem. Furthermore, (3) it will be investigated whether trust in the mother–child relationship and father-child relationship attenuate the association between media pressure and body dissatisfaction and self-esteem. Additionally, (4) it will be investigated whether there are differential associations between trust in the parent–child relationships, their interaction with media pressure, and body dissatisfaction based on the child’s gender. First, consistent with previous research (Nichols et al., 2018; De Coen et al., 2021), it is hypothesized that reporting a higher amount of media pressure will be associated with more body dissatisfaction and less self-esteem. Second, it is assumed that higher levels of trust in parent–child relationships are associated with lower levels of body dissatisfaction and more self-esteem (Smith et al., 2016). Third, based on findings in adolescents (De Vries et al., 2019), it is hypothesized that trust in parent–child relationships will attenuate the association between media pressure and body dissatisfaction and self-esteem. Fourth, in line with the results of the study of De Vries et al. (2019), it is hypothesized that compared with the father-child relationship, especially the mother–child relationship will act as a stronger potential buffer. Although sociocultural influences will examined, certain variables will be controlled for including gender, body mass index (BMI), and pubertal status. While several studies found no gender difference in the prevalence of body dissatisfaction during middle childhood (Dion et al., 2016; Navarro-Patón et al., 2021), other studies found that girls reported more body dissatisfaction than boys (Olive et al., 2012; Neves et al., 2017; Latiff et al., 2018). Furthermore, girls mainly desire a smaller size, linking body dissatisfaction to weight and shape (Wertheim and Paxton, 2011; Dion et al., 2016; Baker et al., 2019; Figueiredo et al., 2019). In contrast, some boys desire a smaller body, while others desire a larger body (Dion et al., 2016; Baker et al., 2019; Figueiredo et al., 2019). BMI has been recognized as potential contributor to the development of body dissatisfaction (Tiggemann, 2005; Lawler and Nixon, 2011; Olive et al., 2012; Baker et al., 2019). Among preadolescent girls, research indicates a positive correlation between increasing BMI and heightened body dissatisfaction, while in preadolescent boys, both low and high BMI levels are linked to such dissatisfaction, forming a U-shaped pattern (Austin et al., 2009). As for puberty status, research showed that girls in early stages of puberty report more body dissatisfaction compared to girls in pre-puberty or later stages of puberty (Mendle, 2014). In contrast, most boys experience increased body satisfaction during puberty due to developing a more muscular, conventionally masculine appearance, reflecting societal ideals (Ricciardelli and McCabe, 2004). In conclusion, it is important to control for these variables when conducting research on body dissatisfaction. Since an association between BMI (van den Berg, 2012) and pubertal status (Voelker et al., 2015) and the outcome variables is expected, the association between adjusted BMI, pubertal status and the outcome variables will be explored and if necessary, adjusted BMI and pubertal status will be included as control variables.

## 2 Method

### 2.1 Participants

A sample of third and fourth grade children was recruited from nine randomly selected elementary schools in the Flemish-speaking region of Belgium. Two weeks before collecting the data, the researchers visited the classrooms and provided an oral explanation to the children about the study’s objectives and procedures. Additionally, information letters were distributed to the children during this visit. Parents received an active informed consent and a letter (both available in six languages to increase inclusion) explaining the purpose and method of the study (“A study using self-report questionnaires about children’s media use, well-being, body image and parent–child relationships”). In total, 635 information letters were handed out to the parents of which 38% gave permission to their children to participate. At the start of the data collection the children were provided with an active informed consent as well, accompanied by a verbal explanation. In total, 246 children (59.8% girls) participated with a mean age of 8.81 years (SD = 0.71; range = 8–10 years).

### 2.2 Procedure

To ensure a clear understanding of the questionnaires among the children, a prior testing of the questionnaire was conducted with a distinct group of several children of the same age. Based on their feedback, certain small modifications were made in order to improve the comprehension of the questionnaire. In order to make it less demanding for the children, the administration of the questionnaires was divided over two timepoints, with a week in between. Each child received a unique code, and filled out the questionnaires during schooltime, in the classroom. Research assistants remained present during the entire period so that they could answer potential questions or, if necessary, read the questions aloud. For each part, children had approximately 60 min to complete the questionnaire. A break of 5 min was provided when the researcher noted that the concentration of the children dropped. After each part the children received a small present and after both parts a group gift for the class was provided. This study was approved by the research ethics committee of the Faculty of Psychology and Educational Sciences of Ghent University.

### 2.3 Measures

#### 2.3.1 Body dissatisfaction

Body dissatisfaction was evaluated using the Appearance subscale of a shortened Dutch version of the Body Esteem Scale for Adolescents and Adults (BESAA) (Mendelson et al., 2001). This subscale consists of six statements designed to measure children’s overall affective assessment of their appearance and bodies, such as “I feel ashamed of how I look.” Participants were required to rate their level of agreement with each statement on a 7-point Likert scale (1 = definitely disagree, 7 = definitely agree). Higher scores on this scale indicate higher levels of body dissatisfaction. The BESAA is based on the body-esteem scale for children (Mendelson and White, 1993). The validity and reliability of the BESAA have been established in Dutch-speaking adolescents (Choi et al., 2021; Prieler et al., 2021) and in both non-Dutch-speaking children (Arslan et al., 2020) and adolescents (Confalonieri et al., 2008; Beltrán-Garrayo et al., 2023), demonstrating good internal consistency and moderate test–retest reliability in children as young as 8 years old. After performing a factor analysis, two items from the BESAA subscale showed corrected item-total correlation values below the commonly accepted threshold of 0.3 (Field, 2009). When looking at “Cronbach’s alpha if item deleted,” our results demonstrated a noteworthy increase in the reliability of the BESAA subscale when these items were omitted. Consequently, these two items (i.e., “I wish I looked like someone else” and “I look as nice as I’d like to be”) were removed and four items (i.e., “There are lots of things I’d change about my looks if I could,” “I wish I looked better,” “My looks upset me” and “I feel ashamed of how I look”) from this subscale were utilized in the analyses to improve the overall reliability (Field, 2009). These items displayed good reliability with a Cronbach’s alpha coefficient of 0.85.

#### 2.3.2 Self-esteem

Self-esteem was measured with the Self-Worth subscale of the Dutch version of the Self-Perception Profile for Children (SPPC; Veerman et al., 1997), designed for children between eight and 12 years old. This subscale consists of six items assessing the feeling of self-worth of the participant, for example “I am happy with myself.” Participants were asked to rate the degree to which they agreed with each statement on a 4-point Likert scale (1 = definitely disagree, 4 = definitely agree). Higher scores indicate a higher feeling of self-worth. Adequate internal consistency, test–retest reliability and discriminant validity were reported in a sample of Dutch-speaking children (Veerman et al., 1996). In the current study, a Cronbach’s alpha of 0.84 was found.

#### 2.3.3 Media pressure

The role of media was evaluated using a Dutch version of the Pressure-Media subscale of the Social Attitudes Towards Appearance Questionnaire-4 (SATAQ 4; Schaefer et al., 2015). This subscale comprises four items that assess the extent to which participants feel pressured by media to conform to societal body ideals, such as “I feel pressure from the media to look in better shape.” Participants were instructed to rate their level of agreement with each statement on a 5-point Likert scale (1 = definitely disagree, 5 = definitely agree). Higher scores indicate a greater perceived pressure to conform to body ideals portrayed in the media. Previous studies in Dutch-speaking children (Trekels and Eggermont, 2018) and in non-Dutch-speaking children (Evans et al., 2013; Neves et al., 2020) demonstrated strong internal reliability and validity. In the current study, a Cronbach’s alpha coefficient of 0.84 was obtained for this subscale.

#### 2.3.4 Trust in parent–child relationships

Trust in parent–child relationships was assessed with the Trust subscale People In My life questionnaire (PIML; Cook et al., 1995), a self-reporting measure, designed for children from 10 to 12 years. The Trust subscale consists of 10 items, which were presented two times, once about the trust in mother and once about the trust in father. Respondents are asked to answer statements using a 4-point Likert scale (1 = Never true, 4 = Always true). Higher scores on this subscale indicate higher levels of trust. Example items include: “My mother/father respects my feelings.” Previous studies in Dutch-speaking children (Bosmans et al., 2019) and in non-Dutch speaking children (Ridenour et al., 2006; Moreira et al., 2017) showed good reliability and adequate construct validity. In the current study, the subscale with items about the mother showed good reliability with a Cronbach’s alpha of 0.84. The subscale with items about the father showed excellent reliability with a Cronbach’s alpha of 0.90.

#### 2.3.5 Adjusted body mass index

The children were requested to provide their weight and height information. The BMI, calculated as weight in kilograms divided by height in meters squared (kg/m^2^), was determined. To enable BMI comparisons across children of different ages and genders, this study employed adjusted BMI values [(actual BMI/50th percentile BMI for age and gender) × 100]. The 50th percentile BMI values for age and gender were established using normative data from a Flemish sample (Roelants and Hauspie, 2004). Based on their adjusted BMI score youngsters can be categorized as underweight (adjusted BMI ≤ 85), normal weight (85 < adjusted BMI < 120), overweight (120 ≤ adjusted BMI <140) or obese (adjusted BMI ≥ 140; Van Winckel and Van Mil, 2001). Research on intraclass correlations between measured and self-reported height, weight and BMI indicate moderately high associations among Belgian children aged 8–11 years old (Himes, 2010).

#### 2.3.6 Pubertal status

Pubertal status was evaluated using the Pubertal Development Scale (PDS) developed by Petersen et al. (1988). This scale assesses five characteristics specific to each sex: growth spurt in height, skin changes, and body hair for both girls and boys, breast development and menarche in girls, and voice changes and facial hair growth in boys. Participants were asked to rate their development on a 4-point Likert (1 = no development, 4 = development already completed). For instance, girls rated items such as “Have your breasts begun to grow?” while boys rated items like “Has your voice begun to change?” For those who had experienced menarche, they were asked to indicate “yes” or “no,” and the age of menarche was recorded. Higher scores on the scale indicate further progress in pubertal development. The PDS has demonstrated reliability in Dutch-speaking children from the age of 8 years old (Koopman-Verhoeff et al., 2020; Waters et al., 2022) and this scale exhibits strong correlations with pubertal stage as assessed by healthcare professionals (Suglia et al., 2020). In the present study, the Cronbach’s alpha coefficient for boys indicated acceptable reliability, with a value of 0.71 for the total score. However, for girls, the Cronbach’s alpha was relatively low, with a value of 0.51.

### 2.4 Statistical analyses

All statistical analyses were performed using SPSS version 26. Prior to conducting the analyses, the data were screened to ensure they met the assumptions of linear regression, including normality, homoscedasticity, and linearity. The dataset had missing data, accounting for 7.51% of the total values. To address this, multiple imputation was employed using the SPSS software, generating five imputations based on the approach outlined by Van der Heijden et al. (2006). This resulted in a final dataset with 0.48% missing data.

To examine differences between boys and girls in the variables of interest, independent *t*-tests were conducted. Furthermore, Pearson correlations were calculated to explore the baseline associations between age, gender, adjusted BMI, pubertal status, media pressure, trust in parent–child relationships, and the outcome measures (body dissatisfaction and self-esteem).

To investigate whether media pressure and trust in parent–child relationships are associated with body dissatisfaction and self-esteem, hierarchical linear regression analyses were used. In order to evaluate the unique contribution of each (group of) independent variables to the dependent variable while controlling for the potential contribution of other independent variables, a step-by-step process was used. The child’s gender (coded as male = 1 and female = 2) and media pressure were entered as independent variables in the first block. Trust in parent–child relationships was included as independent variable in the second block. To investigate whether the trust in parent–child relationships moderates the association between media pressure and body dissatisfaction/self-esteem, the 2-way interaction between trust in parent–child relationships and media pressure was added as independent variable in a third block. Additionally, to examine whether gender moderates these associations, in the fourth block, the 2-way interaction between trust in parent–child relationships and gender, as well as the 3-way interaction between media pressure, trust in parent–child relationships, and gender, were included as independent variables. These analyses were conducted separately for trust in mother and trust in father. To test the moderation models, the SPSS PROCESS macro command set developed by Hayes (2018) was utilized.

## 3 Results

### 3.1 Characteristics of the study sample

Table 1 presents characteristics of the study sample (*n* = 246).

**Table 1:** Characteristics of the study sample (*n* = 246).

	*%/N*	*M*	SD	Range
Age (years)		8.81	0.71	[7–11]
**Grade (N)**				
Third grade	117			
Fourth grade	129			
Girls (%)	59.8			
Adjusted BMI		95.95	19.48	[58.33–178.88]
Underweight (%)	11.5			
Normal weight (%)	80.3			
Overweight (%)	7.5			
Obese (%)	0.7			
Pubertal status		2.47	0.94	[1–5]
Body dissatisfaction		1.89	1.50	[1–5]
Self-esteem		3.47	0.54	[1–4]
Media pressure		1.71	0.98	[1–5]
Trust-mother		3.58	0.48	[1–4]
Trust-father		3.42	0.68	[1–4]

BMI = body mass index; underweight = adjusted BMI ≤ 85; normal weight = 85 < adjusted BMI < 120, overweight = 120 ≤ adjusted BMI < 140; obese = adjusted BMI ≥ 140.

### 3.2 Correlations between the study variables

Table 2 presents baseline correlations between the study variables. Results show that media pressure significantly positively correlates with body dissatisfaction (*r* = 0.25, *p* < 0.001) and significantly negatively with self-esteem (*r* = −0.26, *p* < 0.001). Both trustmother and trust-father significantly negatively correlate with body dissatisfaction (trust-other: *r* = −0.21, *p* = 0.003; trust-father: *r* = −0.21, *p* < 0.001) and significantly positively correlate with self-esteem (trust-mother: *r* = 0.29, *p* < 0.001; trust-father: *r* = 0.24, *p* = 0.001). Age is significantly positively correlated with trust-mother (*r* = 0.24, *p* = 0.001). Adjusted BMI is significantly negatively correlated with trust-mother (*r* = −0.25, *p* = 0.004).

**Table 2:** Pearson correlations between the study variables.

	1	2	3	4	5	6	7
1. Age	–						
2. Adjusted BMI	−0.11	–					
3. Pubertal status	0.02	0.04	–				
4. Body dissatisfaction	0.09	0.16	0.09	–			
5. Self-esteem	0.04	0.12	−0.08	−0.37**	–		
6. Media pressure	−0.05	−0.01	0.15*	0.25***	−0.26***	–	
7. Trust-mother	0.24**	−0.25**	0.08	−0.21**	0.29**	−0.14	–
8. Trust-father	−0.05	−0.04	0.06	−0.28**	0.24**	−0.14	0.29**

BMI = body mass index; *p < 0.05. **p < 0.01. ***p < 0.001.

### 3.3 Exploration of possible control variables

Comparison of gender differences showed no significant differences in age, *t*(224) = 0.04, *p* = 0.972; adjusted BMI, *t*(136) = −1.35, *p* = 0.178; pubertal status, *t*(215) = 0.385, *p* = 0.701, media pressure, *t*(232) = 0.34, *p* = 0.737; trust-mother, *t*(234) = 0.74, *p* = 0.459; trust-father *t*(216) = −0.12, *p* = 0.899 and body dissatisfaction *t*(215) = 0.86, *p* = 0.388. A significant gender difference was found regarding self-esteem, *t*(234) = −2.17, *p* = 0.034, with boys reporting higher levels of self-esteem. Therefore, gender was added as a control variable in the regression analyses. Since adjusted BMI, age and pubertal status are not significantly associated with body dissatisfaction or self-esteem (Table 2), these variables were not included as control variables in the regression analyses.

### 3.4 The association between media pressure and body dissatisfaction and self-esteem

Tables 3, 4 demonstrate the results of the linear regression analyses with body dissatisfaction and self-esteem as outcome variables. In a first block of the regression analysis, gender and media pressure were entered. The results show a significant association between media pressure and both outcome variables, which indicates that children who report a higher amount of media pressure, report higher levels of body dissatisfaction (*β* = 0.35, *p < 0.*001) and lower levels of self-esteem (*β* = −0.26, *p <* 0.001).

**Table 3:** Results from the hierarchical linear regression analyses explaining body dissatisfaction and self-esteem from trust in mother and its interaction with media pressure and gender.

	Body dissatisfaction					Self-esteem				
Variable	*β*	*t*	*p*	*R* ^2^	*ΔR* ^2^	*β*	*t*	*p*	*R* ^2^	*ΔR* ^2^
Block 1				0.06	0.06***				0.09	0.09***
Gender	0.01	−0.15	0.889			0.14	2.08	<0.001		
Media pressure	0.35	3.67	<0.001			−0.26	−3.79	<0.001		
Block 2				0.09	0.03*				0.24	0.15***
Trust-mother	−0.24	−2.55	<0.001			0.39	6.22	<0.001		
Block 3				0.10	0.00				0.24	0.00
Trust-Mother × media pressure	−0.12	−0.68	0.494			−0.14	0.40	0.689		
Block 4				0.11	0.00				0.25	0.01
Trust-mother × gender	−0.16	0.26	0.792			−0.07	−0.12	0.901		
Trust-mother × media pressure × gender	0.17	0.69	0.499			−0.38	−1.66	0.099		

*p < 0.05. ***p < 0.001.

**Table 4:** Results from the hierarchical linear regression analyses explaining body dissatisfaction and self-esteem from trust in father and its interaction with media pressure and gender.

	Body dissatisfaction					Self-esteem				
Variable	*β*	*t*	*p*	*R* ^2^	*ΔR* ^2^	*β*	*t*	*p*	*R* ^2^	*ΔR* ^2^
Block 1				0.09	0.09***				0.10	0.10***
Gender	0.01	0.07	0.946			0.09	1.28	0.201		
Media pressure	0.30	4.17	<0.001			−0.30	−4.25	<0.001		
Block 2				0.14	0.05**				0.15	0.05**
Trust-father	−0.23	−3.24	0.001			0.23	3.22	0.002		
Block 3				0.14	0.00				0.15	0.00
Trust-father × media Pressure	0.12	0.39	0.979			−0.21	−0.72	0.846		
Block 4				0.14	0.00				0.16	0.01
Trust-father × gender	0.15	0.51	0.609			0.64	1.39	0.16		
Trust-father × media pressure × gender	−0.26	−1.11	0.269			−0.24	−0.97	0.335		

**p < 0.01. ***p < 0.001.

### 3.5 Associations between trust in mother and trust in father and body dissatisfaction and self-esteem

In a second block trust-mother (Table 3) and trust-father (Table 4) were added as predictors to investigate whether a positive relationship with their parents was associated with lower levels of body dissatisfaction and higher levels of self-esteem. The results demonstrate that higher levels of trust-mother are related to less body dissatisfaction (*β* = −0.24, *p* < 0.001) and more self-esteem (*β*  = 0.39, *p* < 0.001). Similar results were found for the relationship with the father, as higher levels of trust-father are related to less body dissatisfaction (*β* = −0.23, *p =* 0.001) and more self-esteem (*β* = 0.23, *p* = 0.002).

### 3.6 Trust in mother and trust in father as a protective factor

In a third block the interaction between media pressure and trust-mother (Table 3) and trust-father (Table 4) were added to test whether a positive relationship with their parents can attenuate the relationship between media pressure and body dissatisfaction and self-esteem. Regarding trust-mother, no significant interaction effects on body dissatisfaction (*β* = −0.12, *p* = 0.494) or self-esteem (*β* = −0.14, *p =* 0.689) were found. Comparable results were obtained for the trust-father, as no significant interaction effects on body dissatisfaction (*β* = 0.12, *p* = 0.979) or self-esteem (*β* = −0.21, *p* = 0.846) were found.

### 3.7 Gender of the child

In a fourth block, a two-way interaction between gender and trust-mother/trust-father and a three-way interaction between gender, media pressure and trust-mother/trust-father was added in order to explore whether the previously tested associations are different for boys vs. girls. No significant interactions with gender occurred (all *p*-values > 0.05), indicating that a positive relationship with the mother or father is similarly associated with body dissatisfaction and media pressure in both boys and girls.

## 4 Discussion

This study examined the association between media pressure and children’s body dissatisfaction and self-esteem, and the protective role of the mother–child and father-child relationship herein. Additionally, the differential role of mother–child relationship and father-child relationship was investigated, as well as differences related to children’s gender. Overall, results show that in our sample of elementary school aged children, media pressure was associated with more body dissatisfaction and less self-esteem, while more trust in parent–child relationships was related to less body dissatisfaction and more self-esteem. However, the hypothesis that more trust in parent–child relationships may attenuate the relationship between media pressure and body dissatisfaction and self-esteem was not confirmed. In the following paragraphs, these findings will be further discussed.

First, in contrast to our expectations (van den Berg, 2012; Voelker et al., 2015), BMI and pubertal status were not associated with body dissatisfaction and self-esteem. Consistent with our hypothesis and previous research, our findings show that children who felt more pressure to conform to appearance ideals evaluated their body more negative and felt less secure about themselves (Nichols et al., 2018; De Coen et al., 2021). The perceived pressure to conform to appearance ideals explained 8% of the variance in body dissatisfaction and 7% of the variance in self-esteem during middle childhood, regardless whether or not these appearance ideals have been already internalized. Furthermore, current results indicate that appearance focused media is not only related to body dissatisfaction but also to children’s global self-esteem. Body dissatisfaction and low self-esteem are strongly interrelated and seem to develop concurrently during middle childhood (Harter, 1999; Nichols et al., 2018). This association may be problematic, since appearance focused media emphasizes that one’s self-image should be based on appearance, causing body dissatisfaction and low self-esteem when one is unable to comply with media’s appearance ideals (Grogan, 2016).

Second, in line with a study in preadolescent girls (Smith et al., 2016), the current results showed that more trust in parent–child relationships was associated to less body dissatisfaction and more self-esteem among children, indicating that mutual respect and trust in the relationship between parents and their children is closely intertwined with how children evaluate themselves in general and their body in particular (O'Dea, 2012). These results support the attachment theory as individuals who feel secure in their relationships are more likely to develop a positive model of the self (Bowlby, 1973; Arbona and Power, 2003; Laible et al., 2004; Furman and Buhrmester, 2009). Moreover, individuals with secure attachments tend to experience lower pressure to conform to societal appearance ideals as a means to seek acceptance from others, potentially resulting in reduced body dissatisfaction (Holsen et al., 2012). The findings of the present study support the “interactive” model of body image development, which suggests that the qualities of the parent-relationship, rather than solely parental modeling, play a role in shaping children’s body image (Ogden and Steward, 2000; Smith et al., 2016). Importantly, the current results extend previous findings that mostly focus exclusively on the mother-daughter bond, as it reveals that during middle childhood not only a positive mother–child relationship but also a positive father-child relationship may play a significant role in reducing body dissatisfaction and enhancing self-esteem during middle childhood. Thus, during middle childhood, both mothers and fathers seem to play an important role in children’s body image and self-esteem, while during adolescence a more differentiated picture seems to arise. In the current study, the mother–child relationship explained 3% of the variance in body dissatisfaction and 15% of the variance in self-esteem. Likewise, the father-child relationship explained 5% of the variance in body dissatisfaction and 5% of the variance in self-esteem. The different impact of mothers and fathers can be conceptualized within the independence model, which proposes that all attachment relationships are equally important, but each contribute to development in distinct domains (Bretherton, 2010). Due to the cross-sectional design, no conclusions can be drawn about the direction of these associations and longitudinal research is necessary to gain better understanding.

Third, the present study aimed to investigate whether trust in parent–child relationships can serve as a protective factor against media pressure. Based on a similar study in adolescents (De Vries et al., 2019), it was hypothesized that more trust in parent–child relationships will attenuate the association between media pressure and body dissatisfaction and self-esteem. However, the present results did not support these hypotheses. From a resilience framework, a distinction can be made between promotive and protective parental factors (Masten and Barnes, 2018). Promotive factors are associated with better outcomes at any level of risk, while protective factors are able to reduce the effect of risk factors. Although in the current study, no evidence was found for the role of a trust in parent–child relationships as protective factor, we did find evidence that parental bonds serve as a promotive factor. This indicates that the parent–child relationship appears to be related to body dissatisfaction and self-esteem, regardless of the level of experienced media pressure. In adolescents, researchers did found evidence for the protective role of the mother–child relationship (De Vries et al., 2019). In the current study no relationship was found between trust in parent–child relationships and the level of media pressure, indicating that, at this age, both factors still seem to occur independent from each other. A possible explanation for not finding evidence for a trust in parent–child relationships to moderate the relationship between media pressure and our outcomes, could be that during childhood other, more proximal, parental characteristics interact with media pressure, such as communication about body image or parental modeling of a healthy body image (Handford et al., 2018; Perez et al., 2018). De Vries et al. (2019) examined media exposure, whereas our focus was on media pressure. This distinction raises the possibility that the parent–child relationship may serve as a protective factor against body dissatisfaction in situations involving media exposure but might not offer the same protection when children are already experiencing pressure from media influences. The differences in results may also be due to age differences between participants of both studies. Since levels of media pressure and body image problems tend to increase with age (Bucchianeri et al., 2013; Fardouly and Vartanian, 2016), potentially, the role of protective factors may become more prominent during the developmental period of adolescence. Trust in parent–child relationships might foster the ability to adapt to challenges during adolescence, making differences in resilience more prominent as children age (Orth and Robins, 2014). For instance, prior research has proposed that parents who foster a warm and supportive emotional environment while avoiding coercive tactics are more likely to effectively influence adolescents’ eating behaviors (Lessard et al., 2010). Trust in parent–child relationships diminishes the likelihood that adolescents perceive parental attempts at social control as restrictive, thereby enhancing their freedom. Further research is necessary to gain a comprehensive understanding of the broader impact of various parental characteristics in this context.

Fourth, the findings from the current study indicate that the child’s gender did not moderate the associations between the parent–child relationships and body dissatisfaction or self-esteem. In other words, the relationship with the mother appears to be related not only to the body image and self-esteem of daughters but also of sons, while the relationship with the father appears to be related not only to the body image and self-esteem of sons but also of daughters. These findings are in line with research in adolescents (Keizer et al., 2019) and underscore the need of investigating mother-daughter, mother-son, father-daughter and father-son dyads separately and unravel how mothers and fathers contribute to the development of their children’s body image and self-esteem in the long term.

### 4.1 Strengths, limitations and suggestions for future research

The current study has several strengths. This study may add to theoretical models of body image problems and contributes to sociocultural research on body image in children as it allows to increase insight into potential environmental risk factors, as well as promotive factors. Also, the current study focusses on both boys and girls during middle childhood, which allows to increase insight into the early explanation of body dissatisfaction and into possible gender differences. Since self-report measures were used, a considerable amount of time was spent on reliable measurement of the constructs by reading the questions aloud and giving additional explanations if necessary. Moreover, in contrast to previous research on children’s body image which exclusively focuses on the role of the mother-daughter relationship (Smith et al., 2016), the current research also focused on the role of the father-child relationship. Several limitations have to be considered as well. First, the cross-sectional design of the present study does not allow drawing conclusions on the direction of the effects. A positive parent-relationship may lead to less body dissatisfaction and higher self-esteem but, vice versa, less body dissatisfaction and higher self-esteem may as well result in a more warm family climate and in being less susceptible to media influences. Future work therefore needs to address the developmental course of body dissatisfaction and the role of media variables and the parent–child relationship herein via longitudinal studies. Second, the present study uses a mono-method and mono-reporter design. The data was collected using self-report questionnaires and, thus, only allowing conclusions on trait-related characteristics of body image, self-esteem, the parent–child relationship and media pressure. Other research designs such as ecological momentary assessment designs may increase insight into state body dissatisfaction and unravel momentary associations with risk and protective factors. Also, the addition of parent-reports to measure certain constructs (e.g., parent–child relationship) would be valuable. Third, since this study focuses solely on trust in parent–child relationships, other protective parental characteristics, such as communication and modeling, could be included to examine other potential parental characteristics that may foster resilience. Furthermore, the current study exclusively focusses on parental factors as protective factors, however family influences may also function as a risk factor in the development of body image and eating problems (Van Malderen et al., 2023). Fourth, the percentage of explained variance in the regression models was relatively low, which is possibly due to the complex nature of the outcome variables as they might be influenced by a multitude of variables. Including other sociocultural variables as well as biological and psychological variables might enhance our understanding of body dissatisfaction and low self-esteem (Rousseau et al., 2020). Fifth, the current results do not reveal the reason why there is an association between the parent–child relationship and body dissatisfaction and self-esteem. Research on potential mediating factors would increase insight into the mechanisms underlying the role of the parent–child relationship and its association with body dissatisfaction and self-esteem. For example, researchers argue that individuals who experience their attachment relationship with their parents as more secure, show more adaptive emotion strategies, such as constructive coping (Morris et al., 2007). More adaptive strategies may help children to cope with negative emotions caused by environmental factors, such as media pressure, and, in turn, this may be associated with lower levels of body dissatisfaction and higher levels of self-esteem (Shriver et al., 2021). Future research may also want to investigate whether children with more positive relationships with their parents are less likely to compare themselves with others or internalize current beauty ideals. Finally, not all children knew their height and/or weight, which limited the exploration of adjusted BMI as a predictor. In future research, researchers could directly measure children’s weight and height.

### 4.2 Practical implications

The current results may be of clinical value as they support the idea that the parent–child relationship plays a positive role in boosting children’s body evaluation and self-esteem. Current results carefully indicate that the parents and the emotional climate of the family require attention in prevention and interventions that address body dissatisfaction and self-esteem in children. Professionals can help to promote a secure attachment, for example by focusing on trust and communication in the parent–child relationship. A systematic review has showed that interventions involving parents, that aim to prevent body dissatisfaction and eating disorders in children and adolescents, have led to significant reductions in risk of body image and eating problems (Hart et al., 2015). Furthermore, the current study highlights that fathers can also play a critical part in children’s body-esteem and self-esteem, so they should not be overlooked in intervention programs.

## Data availability statement

The raw data supporting the conclusions of this article will be made available by the authors, without undue reservation.

## Ethics statement

The studies involving humans were approved by Ethics Committee of the Faculty of Psychology and Educational Sciences of Ghent University. The studies were conducted in accordance with the local legislation and institutional requirements. Written informed consent for participation in this study was provided by the participants’ legal guardians/next of kin.

## Author contributions

JC: Conceptualization, Investigation, Methodology, Formal analysis, Visualization, Writing - original draft, Writing - review & editing. SV: Conceptualization, Methodology, Supervision, Writing - review & editing. LG: Conceptualization, Methodology, Supervision, Writing - review & editing.
